# Non-targeted detection of food adulteration using an ensemble machine-learning model

**DOI:** 10.1038/s41598-022-25452-3

**Published:** 2022-12-05

**Authors:** Teresa Chung, Issan Yee San Tam, Nelly Yan Yan Lam, Yanni Yang, Boyang Liu, Billy He, Wengen Li, Jie Xu, Zhigang Yang, Lei Zhang, Jian Nong Cao, Lok-Ting Lau

**Affiliations:** 1 Department of Industrial and Systems Engineering, The Hong Kong Polytechnic University, Hung Hom, Hong Kong China; 2 Research and Innovation Office, The Hong Kong Polytechnic University, Hung Hom, Hong Kong China; 3 Institute for Innovation, Translation and Policy Research, Hong Kong Baptist University, Kowloon Tong, Hong Kong China; 4 Food Safety Consortium, Hong Kong, China; 5 Department of Computing, The Hong Kong Polytechnic University, Hung Hom, Hong Kong China; 6 Inner Mongolia Mengniu Dairy (Group) Co., Ltd, Hohhot, China; 7 Danone Open Science Research Center, Shanghai, China; 8 School of Chinese Medicine, Hong Kong Baptist University, Kowloon Tong, Hong Kong China

**Keywords:** Computational models, Computational platforms and environments, Databases, Machine learning, Programming language, Computational science

## Abstract

Recurrent incidents of economically motivated adulteration have long-lasting and devastating effects on public health, economy, and society. With the current food authentication methods being target-oriented, the lack of an effective methodology to detect unencountered adulterants can lead to the next melamine-like outbreak. In this study, an ensemble machine-learning model that can help detect unprecedented adulteration without looking for specific substances, that is, in a non-targeted approach, is proposed. Using raw milk as an example, the proposed model achieved an accuracy and F1 score of 0.9924 and 0. 0.9913, respectively, when the same type of adulterants was presented in the training data. Cross-validation with spiked contaminants not routinely tested in the food industry and blinded from the training data provided an F1 score of 0.8657. This is the first study that demonstrates the feasibility of non-targeted detection with no a priori knowledge of the presence of certain adulterants using data from standard industrial testing as input. By uncovering discriminative profiling patterns, the ensemble machine-learning model can monitor and flag suspicious samples; this technique can potentially be extended to other food commodities and thus become an important contributor to public food safety.

## Introduction

Economically motivated adulteration (EMA) is an act of intentional food adulteration and a major public health risk^1^. It is an act of deceiving food buyers motivated by economic gains, which contributes significantly to broader issues related to food safety compared with other traditional threats as the contaminants are often unconventional with unknown effects on human health^2^. The human cost of food adulteration amounts to an estimated 75 million foodborne illnesses, 325,000 hospitalisations^3^, and 5,000 deaths per year, and approximately 10% of the food commodities are adulterated according to one study^4^. The Consumer Brands Association (formerly Grocery Manufacturers Association, GMA) estimated the economic loss arising from food fraud to be US$10 billion to US$15 billion per year^5^. Hence, improving the detection of adulteration has both public health and economic benefits. The challenge is that many links in the global food supply chain are at risk of numerous unknown adulterants^6^, threatening food safety and security at a global level.

Some of the food commodities commonly subjected to fraud include olive oil, milk, honey, saffron, and seafood^7^. Because milk is the top target for fraud, EMA of milk and milk products is a serious perennial societal and public health problem. The outbreak of infant formulae tainted with melamine in 2008 demonstrated the severity of human toll caused by food adulteration. With more than 50,000 infants hospitalised and six confirmed deaths^8,9^, studies showed that it was not uncommon for children at that time to be exposed to tainted infant formula^10–12^, causing acute kidney failure, nephrolithiasis, and other urinary abnormalities^13^. By the end of November 2008, 294,000 infants and young children in China were diagnosed with urinary tract stones^9^, and the tally of cases was likely much higher than reported. Moreover, the long-term health effects for those affected are still unknown. By relying on protein specifications, fraudsters adulterated milk protein with nitrogen-rich compounds to make the protein values appear authentic. Other major incidents included the horsemeat scandal in the UK, Ireland, and Europe in 2013, where food advertised as containing beef was found to contain undeclared horse meat; the selling of counterfeit olive oil in Italy in 2009, and the plastic rice scandal, which has affected the global rice supply chain since its emergence in China in 2010^14–16^. Hence, the lack of an effective protocol to detect previously unencountered adulterants can lead to melamine-like outbreaks or fraud in other major food commodities.

Recurrent major fraud incidents related to food in the past decade have drawn the attention of regulatory bodies. Governmental organizations, such as the Food And Drug Administration (FDA) and China National Centre for Food Safety Risk Assessment (CFSA) have been monitoring a wide array of adulterants by different detection methodologies. Large-scale food fraud operations are continuously monitored and seized by the Interpol. The International Food Safety Authorities Network (INFOSAN) was also set up by the Food and Agriculture Organization (FAO) of the United Nations and World Health Organization (WHO) to surveil international food safety.

Despite global collaboration, current detection methods are in effect target-oriented as per the regulations of local legal authorities, that is, the testing ensures that a list of specific substances does not exceed the maximum residual limits. For example, the national standards for raw milk from cows were defined in GB 19,301-2010 in China^17^. Current intervention systems are not designed to determine a near infinite number of potential or previously unencountered contaminants^2^, and the current quality evaluation methods used in industries are often expensive, labour-intensive, and require specialised infrastructure. Therefore, there is an urgent need to develop a non-targeted protocol for effective food surveillance to ensure food safety and security.

Artificial intelligence has been applied successfully to solve many biological problems in a non-targeted manner^18^ because of its ability to integrate vast datasets, learn arbitrarily complex relationships, and incorporate existing knowledge^19^. Compared with traditional chemometric methods, they are more generalisable to the unseen yet retain a high accuracy. Recently, machine-learning methods have been applied to milk authentication. Neto et al. used spectral and compositional data obtained using Fourier transform infrared (FTIR) spectroscopy and machine-learning methods, such as random forest (RF), gradient boosting machine (GBM), and deep-learning convolutional neural network (CNN) architecture to predict the presence of common adulterants in raw milk samples^20^. Piras et al. used matrix-assisted laser desorption/ionization (MALDI) mass spectrometry profiling and machine-learning linear discriminant analysis to determine speciation (cow, goat, sheep, and camel) and milk adulteration^21^. Farah et al. used differential scanning calorimetry and machine-learning methods, such as random forest, GBM, and multilayer perceptron, and multilayer perceptron, to determine the adulteration of raw bovine milk spiked with formaldehyde, whey, urea, and starch^22^. In other food commodities, Gyftokostas et al. used laser induced breakdown spectra and machine-learning algorithms, such as linear discriminant analysis, extremely randomized trees (*ExtraTrees*) classifier, RF classifier, and eXtreme gradient goosting (*XGBoost*) classifier, to predict olive oil authenticity and geographic discrimination^23^. Hu et al. used spectral data from Raman spectroscopy combined with machine-learning algorithms, such as support vector machine, probabilistic neural network, and CNN, to detect adulterated Suichang native honey^24^. *ExtraTrees* and *XGBoost* are two very common machine-learning methods used in food adulteration. *ExtraTrees* is a machine-learning algorithm that consists of multiple decision trees. Compared with RF, *Extratrees* has a high discrimination ability and can be more resilient to noise in the dataset. *XGBoost* is an ensemble learning approach based on classification and regression trees (CART) and is suitable for data with complex structure.

To achieve better food safety, we have developed a non-targeted protocol using a proprietary machine-learning model that can alert and detect any suspicious adulterant without additional testing. The protocol, first tested in milk, could potentially be extended to other food commodities prone to fraud and could become an important contributor for safeguarding food safety and public health by focusing more on intervention than on prevention^2^.

## Results

An archive of 65,632 routinely-tested normal bovine raw milk samples that had passed quality check was provided by Mengniu Dairy Group Co. Ltd. (Menginu) (Helin, Inner Mongolia, China), tested using MilkoScan FT120 (FOSS Analytical, Denmark). Additional 146 samples were intentionally spoilt with cow smell, improperly stored for 36 h, and spiked with different concentrations of potassium sulfate, potassium dichromate, citric acid, and sodium citrate. ammonium sulfate, melamine, urea, lactose, glucose, sucrose, maltodextrin, fructose, water, whole milk powder, skim milk powder, starch, soy milk, and trisodium citrate. Compositional data were available for all samples, comprising eight physiochemical properties: fat, protein, non-fat solid (NFS), total solid (TS), lactose, relative density (RD), freezing point depression (FPD), and acidity. Infrared spectra obtained using the same instrument were available for 372 normal and 285 spiked adulterated samples. Each spectrum was formed by 1056 points measured at wavenumbers ranging from 3000 to 1000 cm^−1^ and were standardized into eight coordinates. Utilization of the full spectra was not recommended owing to the relatively small sample size. Using compositional and absorbance spectral data, individual models of squared Mahalanobis distance (MD) scoring method, *ExtraTrees*, and *XGBoost* and ensemble results of MD, *ExtraTrees*, and *XGBoost* using voting and weighting strategies were investigated to compare the model performance. Squared MD is a commonly used non-decision tree method used in food authentication. *ExtraTrees* and *XGBoost* are two commonly used machine-learning algorithms based on decision trees with high discrimination ability and resilience to noise. Model selection was based on random splitting of the original dataset into training and testing datasets, which achieved the highest F1 score as this score suited the uneven class distribution of the normal and adulterated milk samples in real-life scenarios.

### Machine-learning model using compositional data of raw milk

The compositional data for raw milk (n = 65, 632) and an MD score cutoff of 30.1 (Supplementary Fig. S1.) indicated that *XGBoost* was the best model among MD score, *ExtraTrees*, *XGBoost*, voting, and weighting methods (Supplementary Table S1.), with accuracy and F1 score of 0.9994 and 0.9318, respectively. Among the eight compositional features, RD was the most indicative factor for both *ExtraTrees* and *XGBoost* for detecting potentially adulterated samples (Supplementary Tables 2 and 3 and Supplementary Fig. S2 and S3.).

### Model using selected coordinates from full absorbance spectra

To examine whether selected coordinates from the full absorbance spectra could also be used to authenticate raw milk, the individual and ensemble models were used (n = 657). An MD score cutoff of 1.4 (Supplementary Fig. S4.) indicated that *ExtraTrees* was the best algorithm for the full absorbance spectra of raw milk, with an accuracy and F1 score of 0.972 and 0.9682, respectively (Supplementary Table S4.).

### Model using compositional data and selected coordinates from full absorbance spectra

To further examine whether a combination of compositional data and selected coordinates from the full absorbance spectra can increase the analytical power, individual and ensemble models were used (n = 657 samples). An MD score cutoff of 1.3 (Supplementary Fig. 5) indicated that the weighting method of *ExtraTrees* and *XGBoost* was the best model for the compositional data and selected coordinates from the full absorbance spectra of raw milk, with the highest accuracy and F1 score of 0.9924 and 0.9913, respectively (Supplementary Table 5). Therefore, the weighting method of *ExtraTrees* and *XGBoost* was selected for the subsequent cross-validation.

### Drift effect of raw milk samples

**Figure 1:**
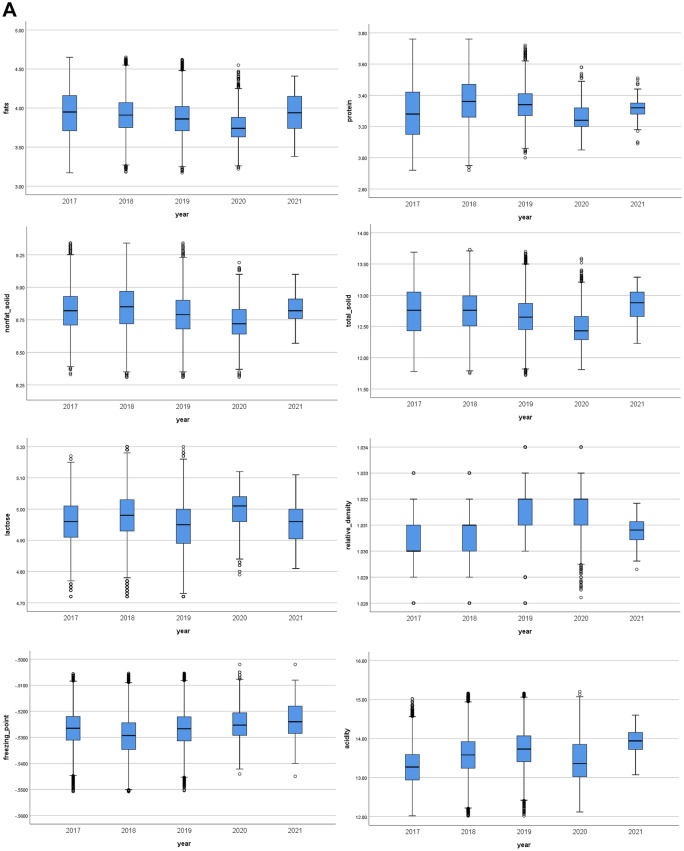

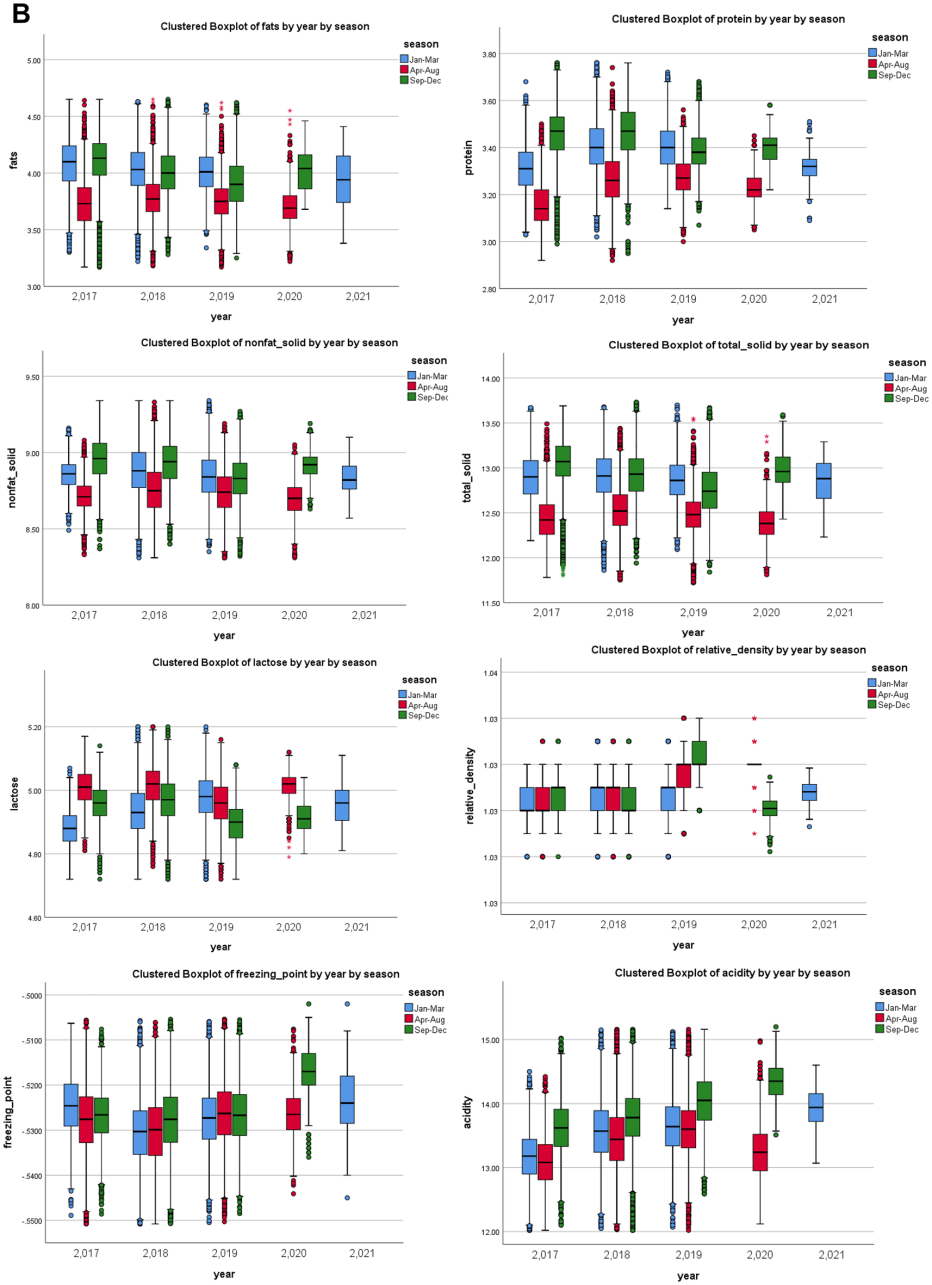
Boxplots of eight compositional features of normal raw milk separated by (**a**) years and (**b**) seasons. Non-fat solid (NFS), total solid (TS), relative density (RD), freezing point depression (FPD).

To assess whether small changes occurring continually over a long period in raw milk can affect the modelling results, drift effects on normal raw milk samples were examined by studying the seasonal or annual variations. Figure 1a shows boxplots of the eight compositional features of normal raw milk separated by years. Statistical analysis showed that all eight compositional features significantly differed for each year (*p-value*: fats; 1.7436E-225, protein; 0.0E0, NFS; 0.0E0, TS; 1.5761E-301, lactose; 0.0E0, RD; 0.0E0, FPD; 0.0E0, and acidity; 0.0E0). Further separation by seasons (January to March, April to August, and September to December) (Fig. 1b) showed that the levels of fats, proteins, NFS, TS, and acidity were lower from April to August and higher from September to December. The opposite effect was observed with lactose. No specific trends were observed for the RD and FPD.

To statistically analyse the presence of such variations, raw milk samples from 2020 (n = 1002, 821 normal and 181 treated) were used to train the models using *XGBoost* (best model for compositional data), and the same trained model was used to predict different samples from 2020 (n = 273, of which 226 were normal and 47 treated) and 2021 (n = 276, 171 normal and 105 treated). A comparison of the models of the raw milk samples from 2020 and 2021 was performed using an independent sample t-test with equal variance, which was not assumed in statistical product and service solutions (SPSS). Statistical analysis showed that the model results for the normal raw milk samples from 2020 were significantly different from those for the normal raw milk samples from 2021, with accuracy, sensitivity, negative predictive value, and F1 score being significantly different between the models (Table 1). This could be attributed to the year-by-year difference in the quality of the raw milk sample or the presence of other operator or machinery variations. Hence, to test the unknown samples, known samples from the same year and batch must be first used to train the model.

**Table 1:** Comparison of the testing data from 2020 (n = 273, of which 226 were normal and 47 treated) with the testing data from 2021 (n = 276). Both were trained by the same model on *XGBoost* using compositional training data from 2020 (n = 1002, of which 821 were normal and 181 were treated). Comparison of the prediction model was performed using independent sample *t-test* on SPSS.

	Accuracy	Sensitivity	Specificity	Precision	Negative predictive value	False alarm	F1 score
2020 testing data	0.9807 ± 0.0055	0.9333 ± 0.0306	0.9912 ± 0.0088	0.9605 ± 0.0392	0.9854 ± 0.0065	0.0088 ± 0.0088	0.946 ± 0.0147
2021 testing data	0.8273 ± 0.0091	0.6381 ± 0.0625	0.9435 ± 0.0338	0.8802 ± 0.0626	0.8102 ± 0.0214	0.0565 ± 0.0338	0.7366 ± 0.0255
Independent samples *t-test p-value*	0.000016	0.002000	0.077000	0.133000	0.000172	0.077000	0.000249

### Cross validation of the selected machine-learning model with blinded samples

Cross-validation was performed using spiked samples blinded from the training data. Model training was performed on the previously available samples with both compositional and spectral data (n = 657, among which 372 were normal raw milk samples and 285 were spiked raw milk samples). Model testing was performed on 65 normal raw milk samples and 90 raw milk samples spiked with hydrogen peroxide (n = 12), sodium hydroxide (n = 15), salt (n = 15), glucose (n = 15), fructose (n = 15), and sucrose (n = 15) with a serial dilution of 0.01, 0.02, 0.05, 0.1, and 0.2 g/100 g of raw milk provided by Mengniu. Hydrogen peroxide, sodium hydroxide, and salt represented new adulterants that were not present in the previous dataset. The compositional data and full absorbance spectra were considered. The weighting method of *ExtraTrees* and *XGBoost* was chosen for modelling the compositional data and selected coordinates from the full absorbance spectra of raw milk.

Upon using the weighting method of *ExtraTrees* and *XGBoost* to model the compositional data and selected coordinates from the full absorbance spectra of raw milk, the modelling performance improved drastically as the drift effect was considered using sugar (glucose, fructose, and sucrose) adulterants (n = 45) from the cross-validation dataset to train the model first. The aforementioned model performed better with sodium hydroxide and/or salt than with hydrogen peroxide, as evident by a drop in the accuracy and F1 score when hydrogen peroxide was being tested or was used to train the model. The best performance was achieved for the prediction of sodium hydroxide and salt when the training samples with sugar adulterants were taken from the cross-validation batch and previous training dataset. In this case, the accuracy, sensitivity, specificity, precision, negative predictive value, false alarm, and F1 score were reported to be 0.9053, 0.9667, 0.8769, 0.6923, 0.8271, 0.1231, and 0.8657, respectively. However, generally, the model performance improved when more training samples were taken from the cross-validation dataset (Table 2).

**Table 2:** Cross-validation with the blind-tested samples and the respective accuracy, sensitivity, specificity, precision, negative predictive value, false alarm, and F1 score. Comparison of drift effect by excluding and including sugar (glucose, fructose, and sucrose) adulterants from the same batch of blind-tested samples in the training dataset. A comparison analysis by including and excluding other adulterants not excluded from the training dataset was also performed. Hydrogen peroxide (H_2_O_2_), sodium hydroxide (NaOH).

	Testing of adulterant(s) blinded to training																			
	Does not consider drift effect							Considers drift effect												
								Training with sugar adulterants, testing of normal raw milk samples from the cross-validation batch							Training with sugar and other adulterants, testing of normal raw milk samples from the cross-validation batch					
	H2O2	NaOH	salt	H2O2, NaOH	H2O2, salt	NaOH, salt	H2O2, NaOH, salt	H2O2	NaOH	salt	H2O2, NaOH	H2O2, salt	NaOH, salt	H2O2, NaOH, salt	H2O2	NaOH	salt	H2O2, NaOH	H2O2, salt	NaOH, salt
Accuracy	0.5120	0.5280	0.5120	0.4714	0.4571	0.4714	0.4258	Undetermined	0.8625	0.8875	0.8316	0.7895	0.9053	0.8182	0.8125	0.8625	0.8500	0.8316	0.8211	0.8947
Sensitivity	0.0500	0.0667	0.0500	0.0533	0.0400	0.0533	0.0444	Undetermined	1.0000	0.9333	0.7333	0.6000	0.9667	0.7333	0.8667	1.0000	1.0000	0.8667	0.7000	1.0000
Specificity	0.9385	0.9538	0.9385	0.9538	0.9385	0.9538	0.9538	Undetermined	0.8308	0.8769	0.8769	0.8769	0.8769	0.8769	0.8000	0.8308	0.8154	0.8154	0.8769	0.8462
Precision	0.4286	0.5714	0.4286	0.5714	0.4286	0.5714	0.5714	Undetermined	0.5769	0.6364	0.7333	0.6923	0.7838	0.8049	0.5000	0.5769	0.5556	0.6842	0.7241	0.7500
Negative predictive value	0.5169	0.5254	0.5169	0.4662	0.4586	0.4662	0.4189	Undetermined	1.0000	0.9828	0.8769	0.8261	0.9828	0.8261	0.9630	1.0000	1.0000	0.9298	0.8636	1.0000
False alarm	0.0615	0.0462	0.0615	0.0462	0.0615	0.0462	0.0462	Undetermined	0.1692	0.1231	0.1231	0.1231	0.1231	0.1231	0.2000	0.1692	0.1846	0.1846	0.1231	0.1538
F1 score	0.0896	0.1194	0.0896	0.0976	0.0732	0.0976	0.0825	Undetermined	0.7317	0.7568	0.7333	0.6429	0.8657	0.7674	0.6341	0.7317	0.7143	0.7647	0.7119	0.8571

Further model testing with each adulterant that was excluded from the model training showed that except for hydrogen peroxide, all adulterants could readily be detected by the system with an F1 score of 0.6364–1.000 and a lower limit of detection (LOD) value of 0.01 g/100 g of raw milk (Table 3). The system was the least sensitive when predicting potassium dichromate, and when lactose was tested, a false alarm could easily be triggered. Similar performances were observed across other models (data not shown). The results demonstrate the feasibility of detecting adulterants in raw milk with high sensitivity, as well as the generalizability of the model in detecting other non-targeted adulterants not included in the training data.

**Table 3:** Model testing with each adulterant excluded from the model training and the respective accuracy, sensitivity, specificity, precision, negative predictive value, false alarm, and F1 score.

	Accuracy	Sensitivity	Specificity	Precision	Negative predictive value	False alarm	F1 score
Ammonium sulfate	1.0000	1.0000	1.0000	1.0000	1.0000	0.0000	1.0000
Citric acid	0.9250	0.6000	1.0000	1.0000	0.9155	0.0000	0.7500
Fructose	1.0000	1.0000	1.0000	1.0000	1.0000	0.0000	1.0000
Glucose	0.9524	0.8667	1.0000	1.0000	0.9310	0.0000	0.9286
Hydrogen peroxide	Undetermined	Undetermined	Undetermined	Undetermined	Undetermined	Undetermined	Undetermined
Lactose	0.8696	0.8667	0.8704	0.6500	0.9592	0.1296	0.7429
Maltodextrin	0.9130	0.6000	1.0000	1.0000	0.9000	0.0000	0.7500
Melamine	0.9048	0.7333	1.0000	1.0000	0.8710	0.0000	0.8462
Sodium hydroxide	1.0000	1.0000	1.0000	1.0000	1.0000	0.0000	1.0000
Potassium dichromate	0.8841	0.4667	1.0000	1.0000	0.8710	0.0000	0.6364
Potassium sulfate	0.9420	0.7333	1.0000	1.0000	0.9310	0.0000	0.8462
Salt	1.0000	1.0000	1.0000	1.0000	1.0000	0.0000	1.0000
Skimmed milk power	1.0000	1.0000	1.0000	1.0000	1.0000	0.0000	1.0000
Soy milk	1.0000	1.0000	1.0000	1.0000	1.0000	0.0000	1.0000
Starch	1.0000	1.0000	1.0000	1.0000	1.0000	0.0000	1.0000
Sucrose	1.0000	1.0000	1.0000	1.0000	1.0000	0.0000	1.0000
Trisodium citrate	1.0000	1.0000	1.0000	1.0000	1.0000	0.0000	1.0000
Urea	0.9565	0.8000	1.0000	1.0000	0.9474	0.0000	0.8889
Water	0.9855	0.9333	1.0000	1.0000	0.9818	0.0000	0.9655
Whey protein	0.9130	0.6000	1.0000	1.0000	0.9000	0.0000	0.7500

### Effect of sample size using compositional features

A range of proportions of the original sample sizes was iterated to examine the relationship between the sample size and predictive power for *ExtraTrees* and *XGBoost*. For both predictive models, the larger the sample size, the more enhanced the predictive power and the smaller the variance (Supplementary Fig. S6 and S7 and Supplementary Tables S6 and S7).

### Performance comparison with the standards of GB 19,301-2010

Concerning the spiked adulterants, the weighting method of *ExtraTrees* and *XGBoost* was effective in identifying raw milk spiked with various buffering reagents or inorganic chemicals, nitrogen-rich adulterants, carbohydrate-based adulterants, and soymilk, as well as in identifying raw milk diluted with water, samples spoilt with cow smell, and samples improperly stored for 36 h. All the compounds were readily detected, and all spiked adulterants were detectable at 1% (w/w) (Table 4). In addition, the weighting method of *ExtraTrees* and *XGBoost* exhibited superior performance to the standards provided in GB 19,301-2010; the weighting method could detect spiked samples not detected by GB 19,301-2010 and had a lower LOD than that reported in GB 19,301-2010 for all spiked adulterants. For the raw milk samples outside the purview of GB 19,301-2010, only one sample spiked with potassium dichromate and another sample that had been improperly stored for 36 h at room temperature were detected as normal by the weighting method (Table 4). Overall, the weighting method of *ExtraTrees* and *XGBoost* exhibited superior performance compared to standards provided in GB 19,301-2010.

**Table 4:** Number of spiked raw milk samples detected by the weighting method of *ExtraTrees* and *XGBoost* and GB 19,301-2010 and the respective lower limit of detection (LOD) for each adulterant. Performance of the weighting method per adulterant was compared with the standard provided in GB 19,301-2010. The weighting method was performed with a 70:30 training to testing ratio, and one iteration was adopted (n = 65,632).

Adulterants	Number of spiked raw milk samples detected by the weighting method of *ExtraTrees* and *XGBoost* (%)	Number of spiked raw milk samples detected by GB 19,301-2010 (%)	LOD of the weighting method of *ExtraTrees* and *XGBoost* (g/100 g of raw milk)	LOD of GB 19,301-2010 (g/100 g of raw milk)	Performance of the weighting method compared to GB 19,301-2010
**Common chemicals**					
Potassium dichromate	13/19 (68%)	4/19 (21%)	0.01	> 0.2	Superior
Potassium sulfate	16/19 (84%)	9/19 (47%)	0.01	0.1	Superior
Sodium citrate	4/4 (100%)	4/4 (100%)	Unknown	Unknown	Same
Trisodium citrate	13/15 (87%)	0/15 (0%)	0.01	> 0.2	Superior
Citric acid	16/19 (84%)	10/19 (53%)	0.01	0.1	Superior
**Nitrogen-based adulterants**					
Ammonium sulfate	14/15 (93%)	5/15 (33%)	0.01	0.1	Superior
Urea	13/15 (87%)	0/15 (0%)	0.01	> 0.2	Superior
Melamine	29/30 (100%)	19/30 (63%)	0.01	0.02	Superior
Whey protein	14/15 (93%)	0/15 (0%)	0.01	> 0.2	Superior
Whole milk powder	14/15 (93%)	0/15 (0%)	0.01	> 0.2	Superior
Skimmed milk powder	14/15 (93%)	0/15 (0%)	0.01	> 0.2	Superior
Soy milk	12/15 (80%)	0/15 (0%)	0.01	> 0.2	Superior
**Carbohydrate-based adulterants**					
Starch	13/15 (87%)	0/15 (0%)	0.01	> 0.2	Superior
Sucrose	14/15 (93%)	0/15 (0%)	0.01	> 0.2	Superior
Glucose	9/15 (60%)	0/15 (0%)	0.01	> 0.2	Superior
Lactose	15/15 (100%)	0/15 (0%)	0.01	> 0.2	Superior
Fructose	13/15 (87%)	0/15 (0%)	0.01	> 0.2	Superior
Maltodextrin	12/15 (80%)	4/15 (27%)	0.01	0.1	Superior
**Others**					
Improperly stored for 36 h	16/23 (70%)	11/23 (48%)	NA	NA	Superior
Water	16/17 (94%)	2/17 (12%)	0.01	> 0.2	Superior
Cow smell	10/10 (100%)	10/10 (100%)	NA	NA	Same

## Discussion

In this study, we described a machine-learning model for testing food adulteration based on an ensemble weighting algorithm of *ExtraTrees* and *XGBoost*. This model achieved F1 scores of 0.9318–0.9913 based on different data types, and cross-validation of raw milk spiked with common, unprecedented contaminants not routinely tested in the industry achieved an F1 score of 0.8657. Even as the food industry continues to amass voluminous testing data over the years, the data remain underutilized. It is recommended that the food industry use the proposed machine-learning model to monitor raw milk samples for suspicious adulteration and anomalies using data from standard testing to prompt further in-depth characterisation. For an authentication method to be practical in the food industry, it is relatively important to maintain a low false-alarm rate, even at the expense of sacrificing accuracy. In addition, this ensemble machine-learning model requires no extra testing effort from the industry. Thus, the proposed system offers numerous advantages in alerting for the presence of any previously un-encountered and non-routinely tested contaminants. Furthermore, upon continuous data feeding and machine learning on the expanding collaborative database, as well as using the full coordinates of the absorbance spectra, which comprise 1,056 data points, the overall accuracies will increase.

Machine learning has been used to solve the problem of food adulteration; however, none of the previous studies have considered the drift effect by training with the present batch of samples, and most studies have not tested samples blinded to the training model. For example, Lim et al. applied deep neural networks to discriminate fatty acid profile patterns between oil mixtures. However, the methodology is impractical as the determination of fatty acid methyl esters (FAMES) profile by a gas chromatography flame ionization detector is expensive and is not routinely performed in the food industry^25,26^. Furthermore, the robustness of the model to newly encountered oil could not be established as only cottonseed oil samples were blended and tested^25^. Neto et al. also evaluated the use of various machine-learning techniques to predict the presence of adulterants in raw milk samples. However, uneven class distribution and drift effects were not considered as 2470 raw and 2376 adulterated milk samples were used in the study. Furthermore, adulterant testing blinded to the training dataset was not performed, challenging the practicability of detecting unknown adulterants. Nevertheless, it could not be ascertained whether the superior performance of the CNN over RF and GBM was a consequence of the machine-learning technique or because different data types were used^14,20,25,26^. Other studies employing machine learning to predict the authenticity of olive oil, milk, honey, and geographical origin of rice used a data acquisition methodology that was impractical in the food industry, drift effect was not considered and did not test samples blinded to the training model^21–24,27^.

The proposed model provides a more practical solution to the food industry by allowing the detection of unprecedented adulterants that have never been encountered. This non-target method with no a priori knowledge of the presence of certain adulterants may minimise the risk of melamine-like incidents in the future. Moreover, the proposed method does not require additional labour-intensive laboratory testing that uses expensive, specialised machinery, thus reducing time and cost while providing extra safeguards. Furthermore, in this method, manually establishing tolerance levels for known authentic reference products was not required as establishing cutoff and decision trees was purely mathematical and computational. Finally, the proposed machine-learning model could identify newly engineered and previously unknown adulterants that have passed the standards of GB 19,301-2010, thus providing extra protection beyond mere fulfillment of the minimum requirement.

Our study is foremost in demonstrating that model performance can be improved drastically when the drift effect is considered during model training with more samples taken from the cross-validation dataset. However, the drop in accuracy and F1 score when hydrogen peroxide is being tested or used to train the model warrants further investigation as it cannot be deduced whether the drop is caused by experiment procedures or the chemical dissociation of hydrogen peroxide. The training dataset must be enlarged and spiked with a more diverse range of adulterants from different seasons. Nevertheless, even with a small sample size of 657 compositional and spectral data, this study managed to demonstrate the feasibility of detecting other non-targeted adulterants excluded from the training data with high sensitivity.

Our study showed that the ensemble decision tree methodologies, *ExtraTrees* and *XGBoost*, outperformed the MD scoring methodology. This is intuitive because *ExtraTrees* randomly selects the splitting values during the splitting of decision trees, which reduces the bias and variance when multiple decision trees are integrated. Through extension to the general loss function, learning with an additive training trick, and overfitting, *XGBoost* possesses better generalisation ability and better handles sparse datasets, with missing values and noise, compared to the other multiple additive regression trees (MART) methods and machine-learning models, such as linear and logistic regression, support vector machines, neural networks, and tree-based models^28–30^.

In addition, our study showed that the model built from the selected coordinates of the full absorbance spectra exhibited superior performance than the model built from the eight compositional features. This suggests that raw measurement data are more representative than compositional data for authentication. It is also shown that seasonal and annual variations exist, resulting in changes in the compositional features. Hence, the drift effect can potentially be used for quality checking in addition to adulteration detection, providing additional quality assurance from an industrial viewpoint.

Continuous updates to the database must be performed on an ongoing basis. Comprehensive information on other key chemometrics, preservatives, antibiotics, pesticides, biological fingerprints, and known characteristics of cattle, including geographic-seasonal-logistic variations, cow breeds, geographical upbringing, feeds, and age must be included. Measurement data can also be extended beyond FTIR to cover other instruments for other food commodities along the entire supply chain. Additionally, this model can be used to detect a mixture of adulterants, and historical data can be combined with data collected continuously from ongoing manufacturing processes. Furthermore, this model can potentially be extended to other food commodities, including solid and liquid food commodities, following the same model selection protocol. Owing to the continuous data feeding and machine learning of the expanding collaborative database, the accuracy of the proposed system can be further improved, and the database can be converted into a valuable unified resource for the industry to assure the food safety of other food commodities.

In conclusion, we reported a machine-learning model that can alert the food industry for potential adulteration in a non-targeted approach without additional testing and used raw milk samples as an example to test the proposed model. By uncovering the discriminative profile patterns, an ensemble machine-learning model can continuously monitor and flag suspicious samples for further in-depth testing. With potential application to other food commodities, the utility of this model and the collaborative database pave the way toward unifying quality standards, assuring food safety, and preventing incidents caused by food adulteration.

## Methods and materials

### Normal and spiked raw milk samples

Archive data of 65,547 normal bovine raw milk samples sampled between 2017 and 2019 were provided by Mengniu and retrieved from in-house laboratory information management systems (LIMS). The data included data from tests that were routinely performed during industrial quality check testing; one such routine testing was performed on MilkoScan FT120 (FOSS Analytical, Denmark) using FTIR spectroscopy. Compositional data from MilkoScan FT120 comprised eight physiochemical properties of the milk samples: fat, protein, NFS, TS, lactose, RD, FPD, and acidity. The numerical values for the different milk components were determined by a series of calculations based on a multiple linear regression (MLR) model that considered the absorbance of light energy by the sample for specific wavelength regions obtained using an FTIR equipment. The readings were performed once. Among the 65,547 raw milk samples, 1,469 (2.21%) were removed including samples that were labelled as “testing in progress”, (normal) samples that were labelled as “fail”, and samples labelled as “pass”, “unlabelled”, or “untreated” but with one or more compositional features that fell outside the range of mean ± 3 standard deviation (SD) based on the three-sigma rule^31^.

Because no real adulterated milk had been found, spiked samples were used to train and test the model. From April to August 2020, 912 raw bovine milk samples were tested by Mengniu using MilkoScan FT120. A total of 27 samples (2.96%), which included samples that were labelled as “unlabelled” but with one or more compositional features that fell outside the range of mean ± 3 SD, were excluded. Among the remaining 885 samples, 834 were normal (94.24%) and 51 (5.76%) were intentionally spoilt with cow smell, improperly stored for 36 h, and spiked with potassium sulfate, potassium dichromate, water, citric acid, and sodium citrate. Table 5 shows the concentrations of the adulterants added. The compositional data (n = 885) were obtained using FTIR spectroscopy, and the readings were performed once.

**Table 5:** Number of spiked samples in 1) model training and selection, 2) cross-validation. All samples were spiked with adulterants with concentrations 0.01, 0.02, 0.05, 0.1, 0.2 g per 100 g raw milk.

Adulterants added	Number of raw milk samples in model training and selection	Number of raw milk samples in cross-validation
**Common chemicals**		
Potassium dichromate	19	0
Potassium sulfate	19	0
Sodium citrate	4	0
Trisodium citrate	15	0
Citric acid	19	0
Hydrogen peroxide	0	15
Sodium hydroxide	0	15
Salt	0	15
**Nitrogen-based adulterants**		
Ammonium sulfate	15	0
Urea	15	0
Melamine	30	0
Skimmed milk powder	15	0
Soy milk	15	0
Urea	15	0
Whey protein	15	0
Whole milk powder	15	0
**Carbohydrate-based adulterants**		
Sucrose	15	15
Glucose	15	15
Lactose	15	0
Fructose	15	15
Maltodextrin	15	0
Starch	15	0
Improperly stored for 36 h	23	0
Water	17	0
Cow smell	10	0
Total	111	90

From September 2020 to February 2021, 770 raw bovine milk samples were tested by Mengniu using FOSS FT120. A total of 113 samples (14.689%), which included samples that were labelled as “unlabelled” but with one or more compositional features falling outside the range of mean ± 3 SD, were removed. Among the 6579 remaining samples, 372 (56.62%) were normal raw milk samples and 2855 (43.38%) were spiked raw milk samples. The spiked raw milk samples included samples spiked potassium sulfate, citric acid, potassium dichromate, ammonium sulfate, melamine, urea, lactose, glucose, sucrose, maltodextrin, fructose, water, whole milk powder, whey protein, skimmed milk powder, starch, soy milk, and trisodium citrate. Table 5 shows the number of samples spiked with the corresponding concentrations of adulterants. The compositional data and full absorbance spectra with a wavenumber range of 1000–3550 cm^−1^ were considered in triplicate. Infrared spectra were obtained using the FTIR technique and were formed by 1056 points measured at wavenumbers ranging from 3000 to 1000 cm^−1^.

In April 2021, 155 raw bovine milk samples were tested by Mengniu using FOSS FT120 and used for cross-validation. A total of 65 (41.93%) samples were normal raw milk samples, and 90 (58.06%) samples were spiked raw milk samples. The spiked raw milk samples included samples spiked with hydrogen peroxide, glucose, sodium hydroxide, salt, fructose, and sucrose. Table 5 shows the number of spiked samples with the corresponding concentrations of adulterants. The compositional data and full absorbance spectra with a wavenumber range of 1000–3550 cm^−1^ were considered in triplicate.

Table 6 presents a summary of the number of normal, spiked, and all raw milk samples in their respective years of sampling. Potassium dichromate, potassium sulfate, and hydrogen peroxide are common chemicals used to increase shelf life; sodium citrate, citric acid, sodium hydroxide, and salt are common chemicals used to maintain correct pH. Nitrogen-based adulterants, such as ammonium sulfate and urea, are used to increase shelf life and volume; while melamine, whey protein, soy milk, and whole and skimmed milk powder are used as diluent to artificially alter the protein content after dilution with water. Carbohydrate-based adulterants, such as starch, sucrose, glucose, lactose, fructose, and maltodextrin are used to increase the carbohydrate content and density of the milk. Finally, water is commonly used as a diluent in milk^32^. All of the abovementioned adulterants are not commonly tested in the dairy industry, and specific tests for these adulterants are not required by the national standard GB 19,301-2010^17^.

**Table 6:** Number of normal raw milk samples and the year of sampling.

Year the normal raw milk was sampled	Number of normal raw milk samples	Number of spiked raw milk samples	Number of all raw milk samples
2017	12,450	0	12,450
2018	28,008	0	28,008
2019	23,602	0	23,602
2020	1047	231	1278
2021	236	195	431

### Standardization of full absorbance spectra into selected coordinates of 7 peaks and 1 average

**Figure 2:**
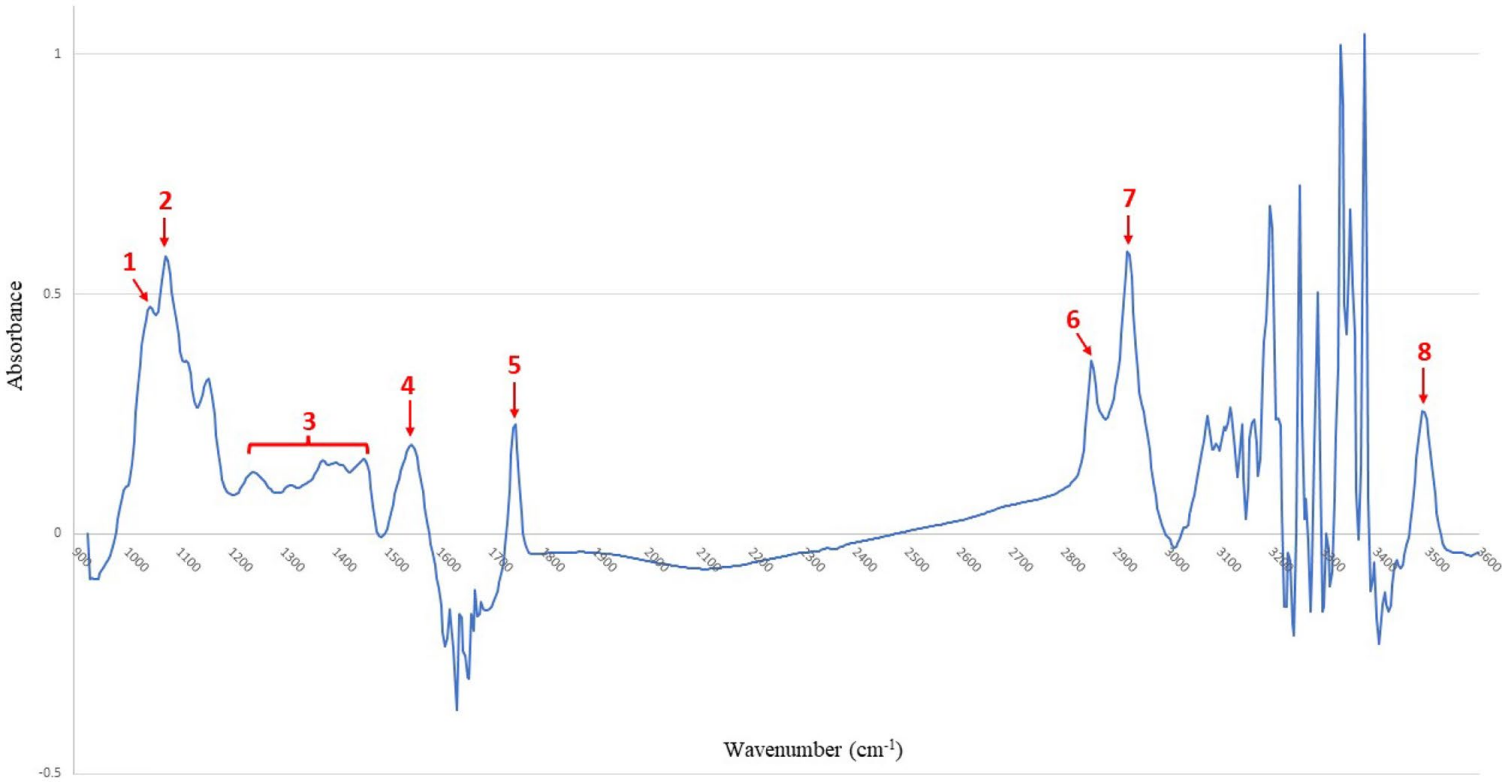
Absorbance spectral data of raw milk retrieved from Fourier transfor infrared (FTIR) spectroscopy in terms of wavenumber. Numbers in red are the eight regions from where the absorbance values were extracted. These include seven peaks within the spectrum regions 1000–1100, 1500–1600, 1730–1800, 2840–2940, 3450–3550 cm^−1^ and an averaged absorbance value of 1250–1450 cm^−1^.

Standardisation of the full absorbance spectra into eight coordinates was performed by the selection of seven peaks within the spectrum regions 1000–1100, 1500–1600, 1730–1800, 2840–2940, and 3450–3550 cm^−1^ and an average absorbance value for 1250–1450 cm^−1^ for each sample (Fig. 2)^33–35^.

### Squared Mahalanobis distance (MD) scoring method

The performances of the decision tree and non-decision tree methods were compared. The MD scoring method is a non-decision tree method used to authenticate raw milk samples. The compositional and absorbance spectral data were used to calculate the squared MD score between each sample and the centroid. Upon iterating a range of MD scores, the MD score with the highest F1 score was considered the MD cutoff for distinguishing atypical from typical raw milk. F1 score considers both false positives and false negatives through the weighted average of precision and recall.

### ExtraTrees

*ExtraTrees* is a machine-learning algorithm proposed by Pierre Geurts *et. al* in 2006 that consists of multiple decision trees^36^. Compared with RF, *Extratrees* has a high discrimination ability and can be more resilient to noise in the dataset because it uses the entire original sample instead of a bootstrap replica to train each decision tree. In this study, we used compositional and spectral data to evaluate how *ExtraTrees* can be used for the binary classification of a sample as typical or atypical^37,38^. The original dataset was randomly split into training and testing datasets. The training dataset was first used to train the *ExtraTrees* predictive model, and the model was verified using a testing dataset to compare the actual and predicted labels. The selection of the best proportion for splitting into the training and test datasets and the number of iterations are discussed in the next section.

### XGBoost

*XGBoost* is an ensemble learning approach based on (CART)^30^. *XGBoost* ensembles trees in a top-down manner. Each tree consists of internal (or split) and terminal (or leaf) nodes. Each split node makes a binary decision, and the final decision is based on the terminal node reached by the input feature. Tree-ensemble methods regard different decision trees as weak learners and then construct a strong learner by either bagging or boosting. Mathematically, the model can be represented by the following objective function with respect to the model parameter $\uptheta :$

$$obj\left( \theta \right) = L\left( \theta \right) + \Omega \left( \theta \right),$$

where $L\left( \theta \right)$ is the empirical loss that must be minimised and $\Omega \left( \theta \right)$ is a regularisation of the model complexity to prevent overfitting. Considering a tree-ensemble model where the overall prediction is the summation of ${\text{K}}$ predictive values across all trees $f_{k} \left( {x_{i} } \right)$,

$${\text{p}}_{{\text{i}}} = \mathop \sum \limits_{{{\text{k}} = 1}}^{{\text{K}}} {\text{f}}_{{\text{k}}} \left( {{\text{x}}_{{\text{i}}} } \right),$$

the objective function can be expressed as:

$${\text{obj}}\left( \theta \right) = \mathop \sum \limits_{i}^{n} l\left( {p_{i} ,t_{i} } \right) + \mathop \sum \limits_{k = 1}^{K} \Omega \left( {f_{k} } \right),$$

where $l\left({p}_{i},{t}_{i}\right)$ is the mean-squared loss imposed on each sample $i$, ${p}_{i}$ is its predictive value, and the labels ${t}_{i}$, $\Omega \left({f}_{k}\right)$ are the regularisation constraints imposed on each tree.

In this study, we used compositional and spectral data to evaluate how *XGBoost* could be used to classify atypical raw milk samples. The original dataset was randomly split into training and testing datasets. The training dataset was first used to train the *XGBoost* predictive model, and the model was predicted using the testing dataset to compare the actual and predicted labels. The two basic hyperparameters, the learning rate of *XGBoost* and maximum depth of the tree, were set empirically at 0.01 and 5°, respectively. The hyper-parameters “min_child_weight” and “col_sample_by_tree” were also tuned carefully with a grid search with tenfold cross-validation, and different seeds were applied in each search process to increase the variance of the model and to find an optimal parameter setting that could maximise the generalisation. For each search iteration, we used the prediction score and calculated the binary cross-entropy with respect to the ground-truth labels, that is, the label indicating whether the testing dataset was normal or spiked. The minimum sum of instance weight (Hessian) required in a child was set to 0.5, the subsample ratio of columns when constructing each tree was set to 0.8, and the objective in specifying the learning task and corresponding learning objective were linear. The hyperparameters “subsample” and “num_boost_weight” required for the selection of the best proportion for splitting into training and test datasets and the number of boosting iterations are discussed in the next section.

### Ensemble model: voting and weighting

The ensemble results of the three methods (MD, *ExtraTrees*, and XGBoost) were investigated to improve the model performance. First, a voting strategy was adopted to combine the results of each method. Training data were used to individually train the MD, *ExtraTrees*, and *XGBoost* models. After obtaining three sets of the initial predicted results from each model, the final predicted result was reported as the majority vote among the three results. The voting strategy was evaluated by comparing the voted result with the label.

In addition to voting, a weighting strategy was adopted. Weights from each of the three methods were assigned based on the individual F1 scores. After training the model for MD, *ExtraTrees*, and *XGBoost* individually, the initial predicted results of the testing data were obtained in binary form (${r}_{1}$, ${r}_{2}$, ${r}_{3}$). The F1 score of each model (${f}_{{m}_{1}}$, ${f}_{{m}_{2}}$, and ${f}_{{m}_{3}}$) and weights (${w}_{1}$, ${w}_{2}$, ${w}_{3}$) were calculated as follows:

$$w_{1} + w_{2} + w_{3} = 1,$$

$$\frac{{f_{{m_{1} }} }}{{w_{1} }} = \frac{{f_{{m_{2} }} }}{{w_{2} }} = \frac{{f_{{m_{3} }} }}{{w_{3} }}$$

The final predicted result calculated as $r = w_{1} r_{1} + w_{2} r_{2} + w_{3} r_{3}$ was evaluated with the labels.

### Selection of the best proportion for splitting into training and test datasets and number of iterations for ExtraTrees and XGBoost

An arbitrary range of proportions for splitting into training and test datasets was examined to determine the optimal proportion of training and testing datasets for *ExtraTrees* and *XGBoost*. Each arbitrary splitting was repeated thrice and with one iteration. The splitting proportions of training-to-testing ratios attempted were 50:50, 60:40, 70:30, 80:20, and 90:10. The proportion with the highest F1 score was selected as the optimal proportion for the corresponding model.

Similarly, a range of iterations was performed to determine the optimal number of iterations for *ExtraTrees* and *XGBoost*. Splitting was performed with the selected proportion of the training and testing datasets, and each splitting was repeated thrice. The iterations were attempted 1, 5, 10, 50, and 100 times. The iteration with the highest F1 score was selected as the optimal iteration for the corresponding model.

The results were reported in terms of accuracy, sensitivity or recall, specificity, precision or positive predictive value, negative predictive value, false alarm, and F1 score. TP, TN, FP, and FN represent true positive, true negative, false positive, and false negative, respectively, and normal raw milk was considered negative whereas spiked raw milk was considered positive.

$${\text{Accuracy}}:\frac{{{\text{TP }} + {\text{ TN}}}}{{{\text{TP}} + {\text{ TN}} + {\text{FP}} + {\text{FN}}}}$$

$${\text{Sensitivity }}\;{\text{or }}\;{\text{recall}}:\frac{{{\text{TP}}}}{{{\text{TP}} + {\text{ FN}}}}$$

$${\text{Specificity}}:\frac{{{\text{TN}}}}{{{\text{FP}} + {\text{ TN}}}}$$

$${\text{Precision }}\;{\text{or }}\;{\text{positive}}\;{\text{ predictive }}\;{\text{value}}:\frac{{{\text{TP}}}}{{{\text{TP}} + {\text{ FP}}}}$$

$${\text{Negative}}\;{\text{ predictive}}\;{\text{ value}}:\frac{{{\text{TN}}}}{{{\text{TN}} + {\text{ FN}}}}$$

$${\text{False}}\;{\text{ alarm}}:\frac{{{\text{FP}}}}{{{\text{FP }} + {\text{ TN}}}}$$

$${\text{F}}_{{1}} \;{\text{score}}:\frac{{{\text{Precision }} \times {\text{recall}}}}{{{\text{Precision }} + {\text{recall}}}} \times 2$$

The model parameters were selected based on the highest F1 scores. In detecting food adulteration, outlier detection implies an unbalanced dataset, with the vast majority of samples being normal raw milk. With such an uneven class distribution, the cost of false positives and false negatives in our dataset can differ significantly. Hence, the F1 score was used instead of accuracy to select the best model as the F1 score considers both false positives and false negatives through the weighted average of precision and recall. The MD calculations, *ExtraTrees*, and *XGBoost* were performed in Python and visualised using PyCharm Community Edition 2021.3.

### Assessment of seasonal and annual variations in raw milk samples

Normal raw milk samples were sub-grouped according to season and year using SPSS. Statistical analysis of annual variations was performed using analysis of variance (ANOVA) with Fisher's least significant difference (LSD) post hoc test. To further examine if the drift effects affected the modelling results, raw milk samples from 2020 (n = 1002, of which 821 were normal and 181 treated) were used to train the models using *XGBoost* (found to be the best model in terms of compositional data), and the same trained model was used to predict different samples from 2020 (n = 273, of which 226 were normal and 47 treated) and 2021 (n = 276, 171 normal and 105 treated). A comparison of the models for the raw milk samples from 2020 and 2021 was performed using an independent sample *t-test* with equal variance, which was not assumed in SPSS. Statistical significance was set at *P* < 0.05.

### Cross validation of the selected machine-learning model with blinded samples

Cross-validation was performed by testing spiked samples blinded from the training data. Model training was performed on previously available samples using both compositional and spectral data (n = 657, of which 372 were normal raw milk samples and 285 were spiked raw milk samples). Model testing were was performed on 65 normal raw milk samples and 90 raw milk samples spiked hydrogen peroxide (n = 12), sodium hydroxide (n = 15), salt (n = 15), glucose (n = 15), fructose (n = 15), and sucrose (n = 15) with serial dilutions of 0.01, 0.02, 0.05, 0.1, and 0.2 g/100 g of raw milk provided by Mengniu. Hydrogen peroxide, sodium hydroxide, and salt represented new adulterants not presented in the previous dataset. The compositional data and full absorbance spectra were both used for the testing. The weighting method of *ExtraTrees* and *XGBoost* was used to model the compositional data and selected coordinates from the full absorbance spectra of raw milk.

To address the problem of drift effect, the inclusion and exclusion of sugar (glucose, fructose, and sucrose) adulterants (n = 45) from the cross-validation dataset into the training dataset were studied and compared. To examine the effect of training the model with more data from the cross-validation dataset, a comparison analysis by including and excluding other adulterants not excluded from the training dataset was also performed. Furthermore, model testing with each adulterant blinded from the training dataset was evaluated.

### Effect of sample size using 8 compositional features

A range of sample sizes was used to determine the relationship between the sample size and predictive power for *ExtraTrees* and *XGBoost*. Each splitting was repeated thrice, with one iteration and a training-to-testing ratio of 90:10. The samples sizes attempted were 20%, 40%, 60%, 80%, and 100% of the original sample size (n = 65, 632).

### Performance comparison to GB 19,301-2010

Each sample was labelled as “pass” or “fail” according to the national standards, as described in *GB 19,301-2010.*

### Ethical approval

None to declare as no human subjects or animal models were required in this study.

## Supplementary Information

Supplementary Information.

## Data Availability

The data that support the findings of this study are available at Mengniu and Danone, which are only authorised for use in the current study and hence are not publicly available. Data are only available upon reasonable request, with written permission from Mengniu and Danone. Requests for data should be made to Prof. Terence Lau.e email address terencelau@hkbu.edu.hk.

## Abbreviations

ANOVA: Analysis of variance
CART: Classification and regression trees
CFSA: China National Centre for Food Safety Risk Assessment
CNNs: Convolutional neural networks
EMA: Economically motivated adulteration
ExtraTree: Extremely randomized trees
FAMES: Fatty acid methyl esters
FAO: Food and Agriculture Organization
FDA: Food and Drug Administration
FN: False negative
FPD: Freezing point depression
FP: False positive
FTIR: Fourier transform infrared
GBM: Gradient boosting machine
GMA: Grocery Manufacturers Association
H_2_O_2_: Hydrogen peroxide
INFOSAN: International Food Safety Authorities Network
LIMS: Laboratory information management systems
LOD: Limit of detection
LSD: Least significant difference
MALDI: Matrix-assisted laser desorption/ionization
MART: Multiple additive regression trees
MD: Squared Mahalanobis distance
Mengniu: Mengniu Dairy Group Co. Ltd.
MLR: Multiple linear regression
NaOH: Sodium hydroxide
NFS: Non-fat solid
RD: Relative density
RF: Random forest
SD: Standard deviation
SPSS: Statistical product and service solutions
TN: True negative
TP: True positive
TS: Total solid
WHO: World Health Organization
XGBoost: EXtreme gradient boosting

## Code availability

The code and model described in this study are available upon reasonable request from Hong Kong Polytechnic University. The request for code access should be made to Prof. Terence Lau. email address terencelau@hkbu.edu.hk. All codes are freely accessible to academic researchers, provided their projects are not supported or involve commercial companies.
