# Supplementary material for: Non-targeted detection of food adulteration using an ensemble machine-learning model
Source: Sci Rep. 2022 Dec 5;12:20956. doi: 10.1038/s41598-022-25452-3 (PMC9722920; doi:10.1038/s41598-022-25452-3)
Supplement: Supplementary file 1 — Supplementary Information. [file 41598_2022_25452_MOESM1_ESM.docx]

**Non-targeted detection of food adulteration using an ensemble machine learning model**

Teresa Chung^1^, Issan Yee San Tam^2^, Nelly Yan Yan Lam^3 4^, Yanni Yang^5^, Boyang Liu^6^, Billy He^5^, Wengen Li^5^, Jie Xu^7^, Zhigang Yang^6^, Lei Zhang^5^, Jian Nong Cao^5^, Lok-Ting Lau ^1,3,4,8^*

1. Department of Industrial and Systems Engineering, The Hong Kong Polytechnic University, Hong Kong

2. Research and Innovation Office, The Hong Kong Polytechnic University, Hong Kong

3. Institute for Innovation, Translation and Policy Research, Hong Kong Baptist University, Hong Kong

4. Food Safety Consortium, Hong Kong

5. Department of Computing, The Hong Kong Polytechnic University, Hong Kong

6. Inner Mongolia Mengniu Dairy (group) Co., Ltd

7. Danone Open Science Research Center, Shanghai, China

8. School of Chinese Medicine, Hong Kong Baptist University, Hong Kong

*Correspondence and requests for materials should be addressed to LT Lau (e-mail: [terencelau@hkbu.edu.hk](mailto:terencelau@hkbu.edu.hk))


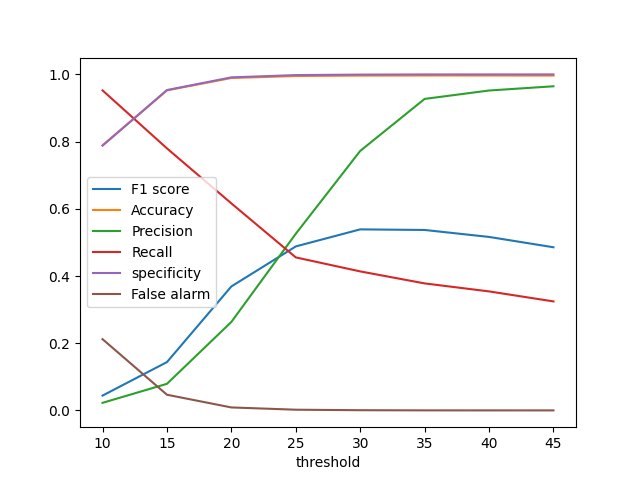


Supplementary Fig. S1. The overall accuracy, sensitivity, specificity, precision, negative predictive value, false alarm, F1 score when iterating a range of squared MD scores for compositional features of raw milk (N=65,632). MD cutoff value selection criteria was the MD score with the highest F1 score.

|  | **Accuracy** | **Sensitivity** | **Specificity** | **Precision** | **Negative predictive value** | **False alarm** | **F1 score** |
| --- | --- | --- | --- | --- | --- | --- | --- |
| MD | 0.9964 | 0.4137 | 0.9994 | 0.7853 | 0.9970 | 0.0006 | 0.5419 |
| *ExtraTrees* | 0.9988 ± 0.0002 | 0.9848 ± 0.0262 | 0.9989 ± 0.0002 | 0.7502 ± 0.0374 | 0.9999 ± 0.0001 | 0.0011 ± 0.0002 | 0.8514 ± 0.0309 |
| *XGBoost* | 0.9994 ± 0.0002 | 0.9318 ± 0.0013 | 0.9997 ± 0.0002 | 0.9322 ± 0.0328 | 0.9997 ± 0 | 0.0003 ± 0.0002 | 0.9318 ± 0.017 |
| Voting method of MD score, *ExtraTrees* and *XGBoost* | 0.9986 ± 0.0004 | 0.7672 ± 0.0408 | 0.9999 ± 0 | 0.9821 ± 0.0015 | 0.9987 ± 0.0004 | 0.0001 ± 0 | 0.861 ± 0.0253 |
| Weighting method of MD score, *ExtraTrees* and *XGBoost* | 0.9987 ± 0.0002 | 0.7253 ± 0.077 | 0.9999 ± 0.0001 | 0.9874 ± 0.0218 | 0.9988 ± 0.0002 | 0.0001 ± 0.0001 | 0.8343 ± 0.0492 |
| Weighting method of MD score and *XGBoost* | 0.9994 ± 0 | 0.9106 ± 0.0281 | 0.9998 ± 0.0001 | 0.9548 ± 0.0232 | 0.9996 ± 0.0001 | 0.0002 ± 0.0001 | 0.9317 ± 0.0069 |
| Weighting method of *ExtraTrees* and *XGBoost* | 0.9994 ± 0 | 0.9106 ± 0.0281 | 0.9998 ± 0.0001 | 0.9548 ± 0.0232 | 0.9996 ± 0.0001 | 0.0002 ± 0.0001 | 0.9317 ± 0.0069 |
| Weighting method of MD score and *ExtraTrees* | 0.9984 ± 0.0002 | 0.7006 ± 0.0384 | 0.9999 ± 0 | 0.9809 ± 0.009 | 0.9985 ± 0.0002 | 0.0001 ± 0 | 0.8171 ± 0.0275 |

Supplementary Table S1. Individual and ensemble model of compositional data of raw milk samples retrieved from the Fourier transform infrared (FTIR) spectroscopy (n=65,632). A MD cutoff of 30.1 was used for compositional data of raw milk. For *ExtraTrees* and *XGBoost*, the optimal % of training and testing ratio and the number of iterations for compositional data for raw milk were achieved with 90:10 training and testing ratio and 1 time iteration respectively (Data not shown). For voting ensemble method and the weighting method of 1) MD score, *ExtraTrees* and *XGBoost*, 2) MD score and XGBoost, 3) *ExtraTrees* and *XGBoost*, the optimal % of training and testing ratio were achieved with 80% training and 20% testing (Data not shown). The optimal % of training and testing ratio of the weighting method for 4) MD score and *ExtraTrees* was achieved with 70% training and 30% testing (Data not shown). For the Weighting method of MD score, *ExtraTrees* and *XGBoost*, relative weight assigned to MD score, *ExtraTrees* and *XGBoost* were 0.2352 ± 0.0198, 0.3568 ± 0.0038 and 0.408 ± 0.0225 respectively. For the Weighting method of MD score and *XGBoost*, the relative weight assigned to MD score and *XGBoost* were 0.3657 ± 0.0322 and 0.6343 ± 0.0322 respectively. For the Weighting method of *ExtraTrees* and *XGBoost*, the relative weight assigned to *ExtraTrees* and *XGBoost* were 0.4668 ± 0.0157 and 0.5332 ± 0.0157 respectively. For the Weighting method of MD score and *ExtraTrees*, the relative weight assigned to MD score and *ExtraTrees* were 0.4084 ± 0.0029 and 0.5916 ± 0.0029 respectively.

|  | **fats** | **protein** | **NFS** | **TS** | **lactose** | **RD** | **FPD** | **acidity** |
| --- | --- | --- | --- | --- | --- | --- | --- | --- |
| **Relative contribution of each feature for *ExtraTrees*** | 0.1469 | 0.0853 | 0.0861 | 0.1427 | 0.0543 | 0.1941 | 0.1427 | 0.1479 |

Supplementary Table S2. Relative contribution of each compositional feature of raw milk samples for the learnt *ExtraTrees* model. Non-fat solid (NFS), total solid (TS), relative density (RD), freezing point depression (FPD).


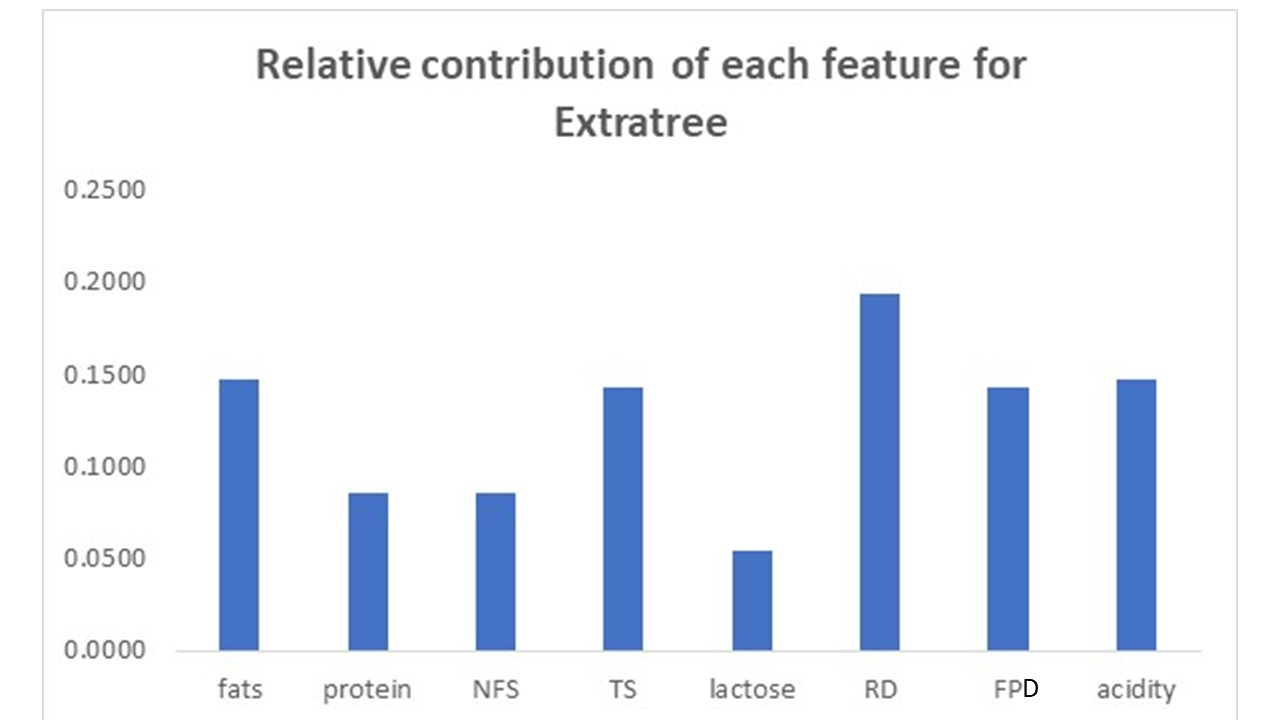


Supplementary Fig. S2. Relative contribution of each compositional feature of raw milk samples for the learnt *ExtraTrees* model. Non-fat solid (NFS), total solid (TS), relative density (RD), freezing point depression (FPD).

|  | **fats** | **protein** | **NFS** | **TS** | **lactose** | **RD** | **FPD** | **acidity** |
| --- | --- | --- | --- | --- | --- | --- | --- | --- |
| **Relative contribution of each feature for *XGBoost*** | 0.0995 | 0.1174 | 0.0940 | 0.0792 | 0.0335 | 0.2859 | 0.1518 | 0.1387 |

Supplementary Table S3. Relative contribution of each compositional feature of raw milk samples for the learnt *XGBoost* model. Non-fat solid (NFS), total solid (TS), relative density (RD), freezing point depression (FPD).


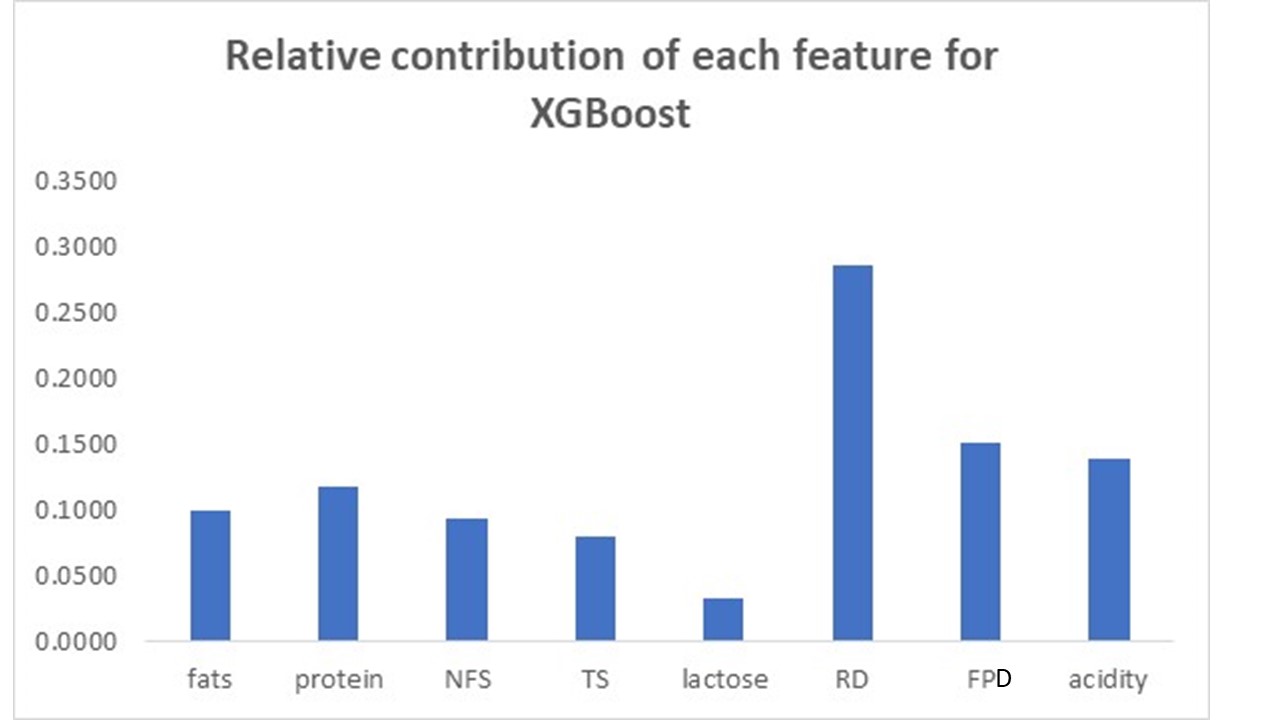


Supplementary Fig. S3. Relative contribution of each compositional feature of raw milk samples for the learnt *XGBoost* model. Non-fat solid (NFS), total solid (TS), relative density (RD), freezing point depression (FPD).


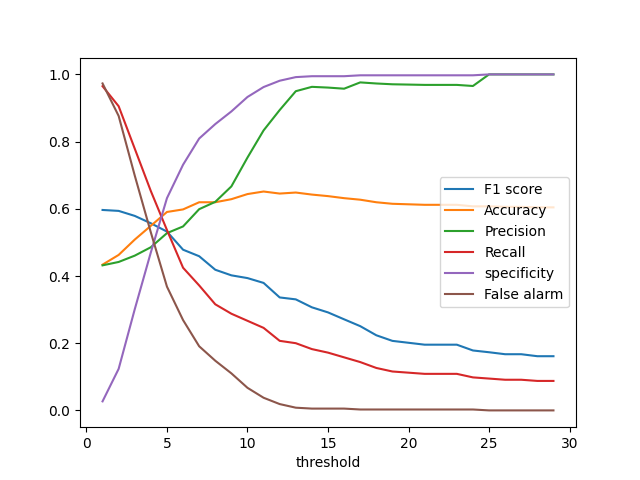


Supplementary Fig. S4. The overall accuracy, sensitivity, specificity, precision, negative predictive value, false alarm, F1 score when iterating a range of squared MD scores for full absorbance spectra of raw milk (N=657). MD cutoff value selection criteria was the MD score with the highest F1 score.

|  | **Accuracy** | **Sensitivity** | **Specificity** | **Precision** | **Negative predictive value** | **False alarm** | **F1 score** |
| --- | --- | --- | --- | --- | --- | --- | --- |
| MD | 0.4460 | 0.9544 | 0.0565 | 0.4366 | 0.6176 | 0.9435 | 0.5991 |
| *ExtraTrees* | 0.972 ± 0.0044 | 0.9599 ± 0.0072 | 0.9815 ± 0.0085 | 0.9768 ± 0.0089 | 0.9681 ± 0.0096 | 0.0185 ± 0.0085 | 0.9682 ± 0.0019 |
| *XGBoost* | 0.9697 ± 0.0303 | 0.9523 ± 0.0414 | 0.9829 ± 0.0296 | 0.9753 ± 0.0428 | 0.9658 ± 0.0296 | 0.0171 ± 0.0296 | 0.9634 ± 0.037 |
| Voting method of MD score, *ExtraTrees* and *XGBoost* | 0.4941 ± 0.0211 | 0.9893 ± 0.0109 | 0.0574 ± 0.0169 | 0.4808 ± 0.019 | 0.8472 ± 0.1684 | 0.9426 ± 0.0169 | 0.6469 ± 0.0171 |
| Weighting method of MD score, *ExtraTrees* and *XGBoost* | 0.9611 ± 0.0178 | 0.9625 ± 0.0341 | 0.9579 ± 0.0198 | 0.9465 ± 0.021 | 0.9741 ± 0.0225 | 0.0421 ± 0.0198 | 0.9542 ± 0.0216 |
| Weighting method of MD score and *XGBoost* | 0.9545 ± 0.0152 | 0.962 ± 0.0371 | 0.949 ± 0.0239 | 0.9286 ± 0.0368 | 0.9748 ± 0.025 | 0.051 ± 0.0239 | 0.9444 ± 0.0218 |
| Weighting method of *ExtraTrees* and *XGBoost* | 0.9712 ± 0.0163 | 0.9544 ± 0.0346 | 0.982 ± 0.0159 | 0.9763 ± 0.0194 | 0.969 ± 0.0194 | 0.018 ± 0.0159 | 0.965 ± 0.021 |
| Weighting method of MD score and *ExtraTrees* | 0.9712 ± 0.0163 | 0.9544 ± 0.0346 | 0.982 ± 0.0159 | 0.9763 ± 0.0194 | 0.969 ± 0.0194 | 0.018 ± 0.0159 | 0.965 ± 0.021 |

Supplementary Table S4. Individual and ensemble model of selected coordinates of full absorbance spectra of raw milk samples retrieved from the Fourier transform infrared (FTIR) spectroscopy (n=657). A MD cutoff of 1.4 was used for spectral data of raw milk. For *ExtraTrees* and *XGBoost*, the optimal % of training and testing ratio for were achieved with 80:20 and 90:10 training and testing ratio. The number of iterations for *ExtraTrees* and *XGBoost* was with 1 time (Data not shown). For voting ensemble method, the weighting method of 1) MD score, *ExtraTrees* and *XGBoost*, 2) MD score and *XGBoost*, 3) *ExtraTrees* and *XGBoost*, and 4) MD score and *ExtraTrees*, the optimal % of training and testing ratio were achieved with 70:30, 70:30, 90:10, 70:30, 70:30 (Data not shown). For the Weighting method of MD score, *ExtraTrees* and *XGBoost*, relative weight assigned to MD score, *ExtraTrees* and *XGBoost* were 0.2375 ± 0.01, 0.3861 ± 0.0041 and 0.3764 ± 0.0074 respectively. For the Weighting method of MD score and *XGBoost*, the relative weight assigned to MD score and *XGBoost* were 0.3788 ± 0.0131 and 0.6212 ± 0.0131 respectively. For the Weighting method of *ExtraTrees* and *XGBoost*, the relative weight assigned to *ExtraTrees* and *XGBoost* were 0.5064 ± 0.0044 and 0.4936 ± 0.0044 respectively. For the Weighting method of MD score and *ExtraTrees*, the relative weight assigned to MD score and *ExtraTrees* were 0.3808 ± 0.012 and 0.6192 ± 0.012 respectively.


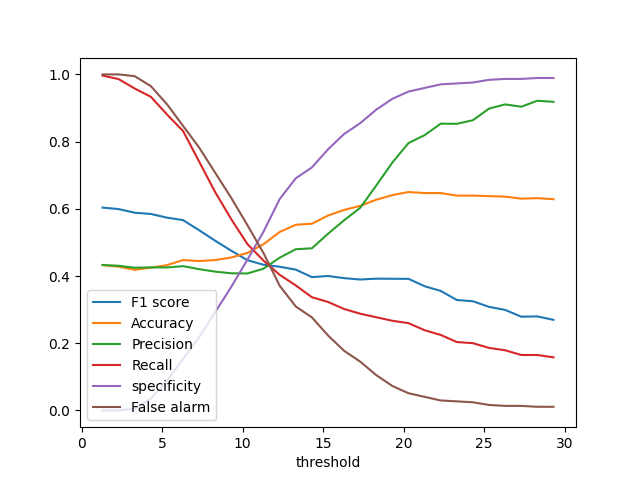


Supplementary Fig. S5. The overall accuracy, sensitivity, specificity, precision, negative predictive value, false alarm, F1 score when iterating a range of squared MD scores for compositional data and selected coordinates of full spectra of raw milk (N=657). MD cutoff value selection criteria was the MD score with the highest F1 score.

|  | **Accuracy** | **Sensitivity** | **Specificity** | **Precision** | **Negative predictive value** | **False alarm** | **F1 score** |
| --- | --- | --- | --- | --- | --- | --- | --- |
| MD | 0.4307 | 0.9930 | 0.0000 | 0.4321 | 0.0000 | 1.0000 | 0.6021 |
| *ExtraTrees* | 0.9873 ± 0.0117 | 0.9736 ± 0.024 | 1 ± 0 | 1 ± 0 | 0.9763 ± 0.0218 | 0 ± 0 | 0.9865 ± 0.0123 |
| *XGBoost* | 0.9746 ± 0.0088 | 0.9652 ± 0.0191 | 0.9813 ± 0.022 | 0.9778 ± 0.024 | 0.9731 ± 0.0119 | 0.0187 ± 0.022 | 0.9713 ± 0.0105 |
| Voting method of MD score, *ExtraTrees* and *XGBoost* | 0.9796 ± 0.0233 | 0.9889 ± 0.0096 | 0.9706 ± 0.0398 | 0.9696 ± 0.0384 | 0.9902 ± 0.0086 | 0.0294 ± 0.0398 | 0.979 ± 0.0229 |
| Weighting method of MD score, *ExtraTrees* and *XGBoost* | 0.9822 ± 0.0117 | 0.9944 ± 0.0098 | 0.9729 ± 0.0147 | 0.966 ± 0.0148 | 0.9952 ± 0.0082 | 0.0271 ± 0.0147 | 0.9799 ± 0.012 |
| Weighting method of MD score and *XGBoost* | 0.9822 ± 0.0117 | 0.9944 ± 0.0098 | 0.9729 ± 0.0147 | 0.966 ± 0.0148 | 0.9952 ± 0.0082 | 0.0271 ± 0.0147 | 0.9799 ± 0.012 |
| Weighting method of *ExtraTrees* and *XGBoost* | 0.9924 ± 0.0132 | 0.9831 ± 0.0294 | 1 ± 0 | 1 ± 0 | 0.9867 ± 0.0231 | 0 ± 0 | 0.9913 ± 0.0151 |
| Weighting method of MD score and *ExtraTrees* | 0.9924 ± 0.0132 | 0.9831 ± 0.0294 | 1 ± 0 | 1 ± 0 | 0.9867 ± 0.0231 | 0 ± 0 | 0.9913 ± 0.0151 |

Supplementary Table S5. Individual and ensemble model of compositional data and selected coordinates of full absorbance spectra of raw milk samples retrieved from the Fourier transform infrared (FTIR) spectroscopy (n=657). A MD cutoff of 1.3 was used for the combination of compositional and spectral data of raw milk. For *ExtraTrees* and *XGBoost*, the optimal % of training and testing ratio for were achieved with 80:20 training and testing ratio. The number of iterations for *ExtraTrees* and *XGBoost* was with 1 time (Data not shown). For voting ensemble method, the weighting method of 1) MD score, *ExtraTrees* and *XGBoost*, 2) MD score and *XGBoost*, 3) *ExtraTrees* and *XGBoost*, and 4) MD score and *ExtraTrees*, the optimal % of training and testing ratio were all achieved with 80:20 (Data not shown). For the Weighting method of MD score, *ExtraTrees* and *XGBoost*, the relative weight assigned to MD score, *ExtraTrees* and *XGBoost* were 0.2313 ± 0.0123, 0.3866 ± 0.0061 and 0.3821 ± 0.0063 respectively. For the Weighting method of MD score and *XGBoost*, the relative weight assigned to MD score and *XGBoost* were 0.377 ± 0.0164 and 0.623 ± 0.0164respectively. For the Weighting method of *ExtraTrees* and *XGBoost*, the relative weight assigned to *ExtraTrees* and *XGBoost* were 0.5029 ± 0.0012 and 0.4971 ± 0.0012 respectively. For the Weighting method of MD score and *ExtraTrees*, the relative weight assigned to MD score and *ExtraTrees* were 0.3743 ± 0.0162 and 0.6257 ± 0.0162 respectively.

Supplementary Fig. S6. Mean and SD of accuracy, sensitivity, specificity, precision, negative predictive value, false alarm, F1 score of the different proportion of the original sample sizes (n=65,632) of the *ExtraTrees* model using compositional data, n=3, with splitting proportion of 90% training dataset and 10% testing dataset and x1 iteration.

| **% of original sample** | **Accuracy** | **Sensitivity** | **Specificity** | **Precision** | **Negative predictive value** | **False alarm** | **F1 score** |
| --- | --- | --- | --- | --- | --- | --- | --- |
| 0.2 | 0.998 ± 0.0016 | 1 ± 0 | 0.998 ± 0.0016 | 0.4778 ± 0.2715 | 1 ± 0 | 0.002 ± 0.0016 | 0.6119 ± 0.2836 |
| 0.4 | 0.9972 ± 0.001 | 1 ± 0 | 0.9972 ± 0.001 | 0.4167 ± 0.0722 | 1 ± 0 | 0.0028 ± 0.001 | 0.5859 ± 0.07 |
| 0.6 | 0.9981 ± 0.0006 | 1 ± 0 | 0.9981 ± 0.0006 | 0.6635 ± 0.0968 | 1 ± 0 | 0.0019 ± 0.0006 | 0.7951 ± 0.0683 |
| 0.8 | 0.9982 ± 0.0004 | 0.9744 ± 0.0444 | 0.9982 ± 0.0004 | 0.6365 ± 0.0927 | 0.9999 ± 0.0001 | 0.0018 ± 0.0004 | 0.7686 ± 0.0796 |
| 1 | 0.9988 ± 0.0002 | 0.9848 ± 0.0262 | 0.9989 ± 0.0002 | 0.7502 ± 0.0374 | 0.9999 ± 0.0001 | 0.0011 ± 0.0002 | 0.8514 ± 0.0309 |

Supplementary Table S6. Mean and SD of accuracy, sensitivity, specificity, precision, negative predictive value, false alarm, F1 score of the different proportion of the original sample sizes (n=65,632) of the *ExtraTrees* model using compositional data, n=3, with splitting proportion of 90% training dataset and 10% testing dataset and x1 iteration.

Supplementary Fig. S7. Mean and SD of accuracy, sensitivity, specificity, precision, negative predictive value, false alarm, F1 score of the different proportion of the original sample sizes (n=65,632) of the *XGBoost*  model using compositional data, n=3, with splitting proportion of 90% training dataset and 10% testing dataset and x1 iteration.

| **% of original sample** | **Accuracy** | **Sensitivity** | **Specificity** | **Precision** | **Negative predictive value** | **False alarm** | **F1 score** |
| --- | --- | --- | --- | --- | --- | --- | --- |
| 0.2 | 0.997 ± 0.0015 | 0.5317 ± 0.1222 | 0.9995 ± 0.0009 | 0.8889 ± 0.1924 | 0.9975 ± 0.0009 | 0.0005 ± 0.0009 | 0.6571 ± 0.1245 |
| 0.4 | 0.9981 ± 0.0008 | 0.7631 ± 0.0972 | 0.9996 ± 0 | 0.9253 ± 0.014 | 0.9985 ± 0.0008 | 0.0004 ± 0 | 0.8344 ± 0.0596 |
| 0.6 | 0.9981 ± 0.0008 | 0.7345 ± 0.0784 | 1 ± 0 | 1 ± 0 | 0.998 ± 0.0008 | 0 ± 0 | 0.8453 ± 0.0534 |
| 0.8 | 0.9989 ± 0.0006 | 0.7813 ± 0.0534 | 0.9999 ± 0.0001 | 0.9861 ± 0.0241 | 0.9989 ± 0.0005 | 0.0001 ± 0.0001 | 0.8714 ± 0.0395 |
| 1 | 0.9994 ± 0.0002 | 0.9318 ± 0.0013 | 0.9997 ± 0.0002 | 0.9322 ± 0.0328 | 0.9997 ± 0 | 0.0003 ± 0.0002 | 0.9318 ± 0.017 |

Supplementary Table S7. Mean and SD of accuracy, sensitivity, specificity, precision, negative predictive value, false alarm, F1 score of the different proportion of the original sample sizes (n=65,632) of the *XGBoost*  model using compositional data, n=3, with splitting proportion of 90% training dataset and 10% testing dataset and x1 iteration.
